# Observational constraints from global ice-phase fraction indicate moderate climate sensitivity

**DOI:** 10.1126/sciadv.aea0731

**Published:** 2026-06-05

**Authors:** Rui Zhou, Tingfeng Dou, Chen Zhou, Ivy Tan, Deliang Chen, Gaojie Xu, Yifan Yang, Xianda Gong, Xiaoming Ju, Aihui Wang, Cunde Xiao

**Affiliations:** ^1^ College of Resources and Environment, University of Chinese Academy of Sciences, Beijing 101408, China.; ^2^ State Key Laboratory of Earth System Numerical Modeling and Application, Chinese Academy of Sciences, Beijing 100864, China.; ^3^ School of Atmospheric Sciences, Nanjing University, Nanjing 210023, China.; ^4^ Joint International Research Laboratory of Atmospheric and Earth System Sciences and Institute for Climate and Global Change Research, Nanjing University, Nanjing 210023, China.; ^5^ Department of Physics, University of Colorado Boulder, Boulder, CO 80309, USA.; ^6^ Department of Earth System Sciences, Tsinghua University, Beijing 100084, China.; ^7^ Research Center for Industries of the Future, School of Engineering, Westlake University, Hangzhou 310030, China.; ^8^ Key Laboratory of Coastal Environment and Resources of Zhejiang Province, School of Engineering, Westlake University, Hangzhou 310030, China.; ^9^ Center for Monsoon System Research, Institute of Atmospheric Physics, Chinese Academy of Sciences, Beijing 100029, China.; ^10^ State Key Laboratory of Earth Surface Processes and Disaster Risk Reduction, Beijing Normal University, Beijing 100875, China.

## Abstract

Cloud feedback remains the largest source of uncertainty in estimates of climate sensitivity. Among its components, the cloud optical depth (τ) feedback acts as a negative feedback that suppresses climate warming, yet it remains poorly constrained by observations. In this study, we constrain the τ feedback in CMIP6 models using global satellite observations of ice-phase fraction from active sensors (CloudSat), passive sensors (MODIS), and a combined active-passive dataset (DARDAR-MODIS). Using the DARDAR-MODIS constraint, the shortwave (SW) τ feedback is updated from the CMIP6 multimodel mean of −0.18 ± 0.14 to −0.43 ± 0.12 watts per square meter per kelvin. This estimate is comparable to, but slightly weaker than, constraints derived independently from MODIS (−0.58 ± 0.17 watts per square meter per kelvin) and CloudSat (−0.46 ± 0.16 watts per square meter per kelvin). Applying Bayesian estimation based on the DARDAR-MODIS–constrained τ feedback, we update the likely range of equilibrium climate sensitivity from [2.6 to 4.0] kelvin to [2.5 to 3.7] kelvin, with the median value decreasing from 3.2 to 3.0 kelvin. The consistency of constraints across multiple satellite datasets suggests a moderate climate sensitivity.

## Teaser

Global observational constraints from ice-fraction advance the understanding of climate sensitivity and inform future projections.

## INTRODUCTION

Climate sensitivity—the equilibrium global mean surface temperature increase following a doubling of atmospheric CO_2_—reflects our fundamental understanding of climate change (*1*–*3*). Among the contributors to uncertainty in climate sensitivity estimates, cloud feedbacks remain the largest source (*4*, *5*). This is largely due to limitations in current observational capabilities for cloud properties and radiative fluxes (*6*), as well as the relatively short duration of reliable satellite records (*7*). As a result, it remains challenging to constrain cloud feedback strength using observations alone. In this context, climate models continue to serve as an essential tool for quantifying different types of cloud feedbacks. However, many models struggle to accurately simulate how cloud properties such as coverage and optical depth respond to warming, introducing substantial uncertainty into cloud feedback estimates (*8*–*10*).

A common approach to reducing this uncertainty is to apply observational constraints to individual components of cloud feedback across different regions. These components include cloud amount, altitude, and optical depth (τ) feedbacks (*11*–*13*). Considerable progress has been made in constraining cloud amount and altitude feedbacks using satellite observations (*14*–*18*). For example, data from CloudSat-CALIPSO and Moderate Resolution Imaging Spectroradiometer (MODIS) have been used to constrain marine low-level cloud feedbacks in tropical and mid-latitude regions (*14*, *15*), while DARDAR-Cloud and 2C-ICE observations have helped constrain tropical anvil cloud area feedbacks (*16*–*17*). CALIPSO observations have also been used to constrain high-cloud altitude feedbacks (*18*, *19*). A summary of these studies is provided in table S1.

In contrast, τ feedback has received far less attention. While several studies have examined τ feedback in low-level clouds over mid- to high-latitude regions (*20*–*24*), no studies to date have quantified τ feedback across the full atmospheric column on a global scale. This study aims to fill that gap by providing observational constraint on τ feedback for clouds at all levels globally.

The ice-phase fraction of cloud condensate plays a key role in determining τ feedback in mixed-phase clouds. For a given amount of condensate, clouds with a higher proportion of ice tend to have a lower optical depth (*10*, *22*, *25*, *26*) and thus reflect less shortwave (SW) radiation back to space. This mechanism also applies when ice and liquid clouds coexist within a vertical column: A higher frequency of ice clouds combined with a lower frequency of liquid clouds yields a smaller total τ, leading to reduced SW reflection (*27*, *28*). To characterize this behavior, we use the cloud ice-to-water ratio (*R*_CI2W_)—defined as the ratio of cloud ice water path (IWP) to total cloud water path—as a metric of the ice-liquid phase distribution and link it to the global SW τ feedback.

We begin by comparing satellite-based *R*_CI2W_ observations with outputs from CMIP6 climate models to assess model performance, revealing a systematic underestimation of *R*_CI2W_ in the models. Models with larger underestimations of *R*_CI2W_ simulate weaker SW τ feedback. This relationship is evident in both spatial patterns and intermodel correlations and is supported by a physically interpretable mechanism: As temperature rises, *R*_CI2W_ decreases and cloud regimes shift toward optically thicker clouds. These clouds reflect more SW radiation, thereby acting as a negative feedback. In this context, a higher climatological *R*_CI2W_ implies greater potential for transitions from optically thin to optically thick clouds, corresponding to a stronger negative SW τ feedback.

Building on this relationship, we apply an emergent constraint approach using satellite-observed *R*_CI2W_ from active sensors, passive sensors, and their combination to correct model biases and refine estimates of the SW τ feedback. Observational uncertainties are explicitly quantified within the emergent constraint framework for each dataset. The constrained feedback estimates are then incorporated into a Bayesian framework to update the likely range of climate sensitivity. Last, we assess how discrepancies among different *R*_CI2W_ observations contribute to uncertainty in the constrained results.

## RESULTS

**Fig. 1.**
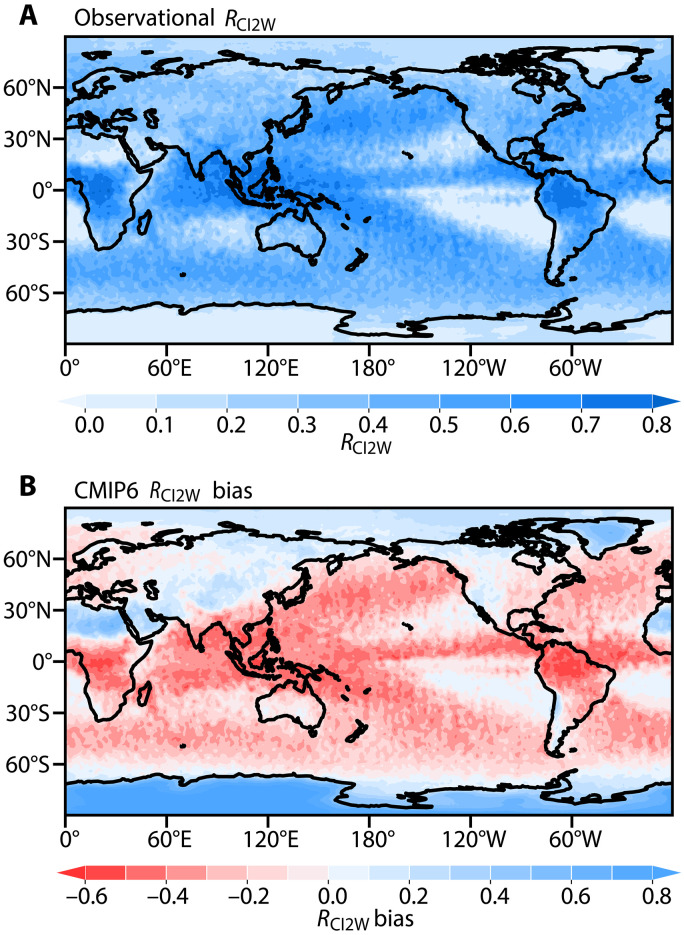
Observed spatial distribution of the *R*_CI2W_ and its multimodel simulation bias. (**A**) Spatial distribution of *R*_CI2W_ derived from the DARDAR-MODIS observations. (**B**) Bias in the CMIP6 multimodel mean *R*_CI2W_ relative to the DARDAR-MODIS observations; negative values indicate regions where models underestimate *R*_CI2W_.

Both CloudSat and MODIS products provide estimates of ice and liquid water paths (IWP and LWP), yet each has distinct limitations. Passive sensors rely on visible-infrared and microwave measurements to retrieve LWP, but they tend to overestimate ice by misclassifying low-level liquid water as ice, particularly in multilayer cloud systems (*29*, *30*). Active sensors (cloud radar and lidar) can penetrate cloud layers and provide more reliable IWP retrievals via a linear temperature-dependent phase partitioning scheme (*31*, *32*); however, they tend to underestimate LWP because of strong signal attenuation, especially in precipitating conditions (*33*, *34*). By integrating active and passive products, we construct a more reliable *R*_CI2W_ dataset (hereafter DARDAR-MODIS; see Materials and Methods) that leverages the complementary strengths of both techniques. In the following analysis, we primarily use DARDAR-MODIS observations (Fig. 1A). To assess the sensitivity of our results to observational uncertainty, we also apply the same constraint framework to two additional independent datasets (MODIS and CloudSat) for comparison (fig. S1).

We first evaluate the performance of CMIP6 models in simulating *R*_CI2W_ using the DARDAR-MODIS observations. The results show that the CMIP6 multimodel mean underestimates *R*_CI2W_ in more than 63% of global regions, with particularly pronounced underestimation over oceanic areas. This indicates a systematic negative bias in simulated global *R*_CI2W_ across models, despite localized overestimations in Greenland, continental Asia, and Antarctica (Fig. 1B and fig. S2). Independent MODIS and CloudSat observations consistently show similar underestimations of *R*_CI2W_ in CMIP6 models, although the magnitude and spatial extent of the bias differ among datasets (fig. S3).

**Fig. 2.**
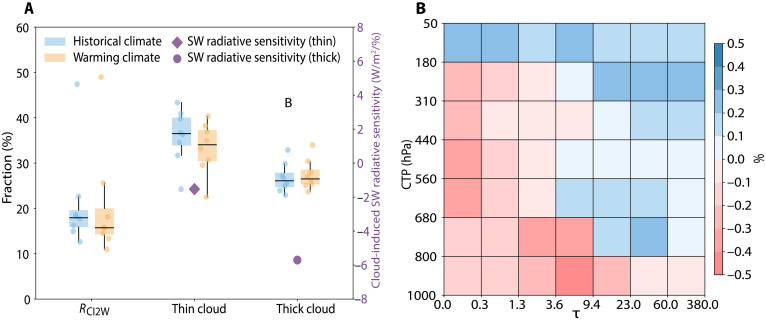
Response of the *R*_CI2W_ and cloud fraction to a 4K climate warming. (**A**) Box-and-whisker plots showing the distributions of *R*_CI2W_ (scaled to percentage by multiplying by 100, %), thin-cloud fraction (%), and thick-cloud fraction (%) under historical (blue) and warmer (orange) climates. The dimensionless *R*_CI2W_ is expressed as a percentage for visualization consistency. Horizontal black lines denote median values, box boundaries indicate the 25th and 75th percentiles, whiskers extend to 1.5 times the interquartile range, and individual climate models are shown as scattered dots. The secondary *y* axis (right, green) shows the cloud-induced SW radiative response per unit change in cloud fraction (W m^−2^ %^−1^) for thin (purple diamonds) and thick (purple circles) clouds. (**B**) Histogram of changes in cloud fraction relative to the historical climate in the joint distribution of cloud optical depth (τ; *x* axis) and cloud-top pressure (CTP; *y* axis, hPa). Red shading indicates decreases in cloud fraction, while blue shading indicates increases.

The widespread underestimation of *R*_CI2W_ suggests that current climate models may systematically underestimate the strength of the SW cloud optical depth (τ) feedback. As temperatures rise, *R*_CI2W_ decreases, driving a regime shift toward optically thicker clouds, particularly in the mid- to upper troposphere (cloud-top pressure < 680 hPa) (Fig. 2). This shift reduces the fraction of optically thin clouds while increasing the fraction of optically thick clouds (fig. S4), thereby enhancing SW reflection and producing a negative feedback. Models with lower *R*_CI2W_ simulate smaller increases in thick-cloud occurrence, which limits the enhancement of column-integrated τ and SW reflection, resulting in a weaker SW τ feedback.

**Fig. 3.**
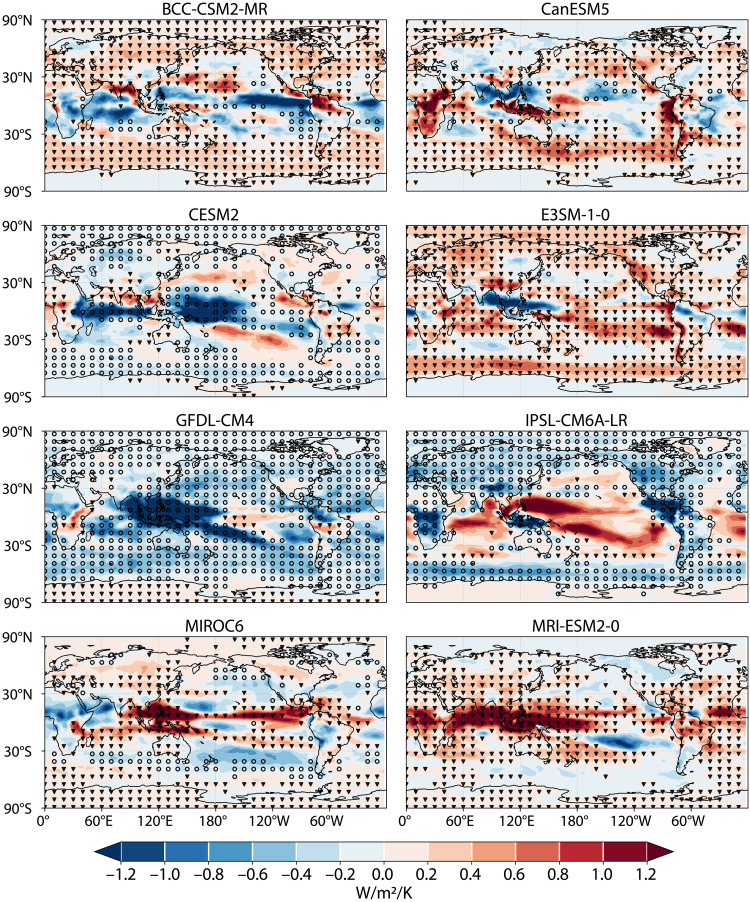
Spatial distribution of anomalies in SW cloud optical depth (SW τ) feedback relative to the multimodel mean. Triangles mark regions where lower *R*_CI2W_ is associated with weaker SW τ feedback, while open circles indicate regions where higher *R*_CI2W_ corresponds to stronger SW τ feedback. The markers highlight the spatial correspondence between *R*_CI2W_ and SW τ feedback strength across models.

The relationship between *R*_CI2W_ and SW τ feedback is evident not only in the global mean but also across different regions (Fig. 3). Despite substantial intermodel spread in feedback patterns, BCC-CSM2-MR, CanESM5, E3SM-1-0, and MRI-ESM2-0 simulate weaker SW τ feedback (red anomalies) coincident with lower *R*_CI2W_, whereas CESM2, GFDL-CM4, and IPSL-CM6A-LR exhibit stronger feedback (blue anomalies) associated with higher *R*_CI2W_ (Fig. 3). This indicates that the simulated ice-to-water ratio within the vertical atmospheric column plays a key role in determining the strength of the SW τ feedback in current climate models.

Further analysis shows that the global SW τ feedback is driven primarily by mid- to high-level clouds (fig. S5). In particular, the underestimation of *R*_CI2W_ over mid- to low-latitude oceans—where *R*_CI2W_ exhibits a strong negative correlation with the feedback from these cloud levels (*r* = −0.96, *P* < 0.001)—propagates to influence the global τ feedback estimate.

**Fig. 4.**
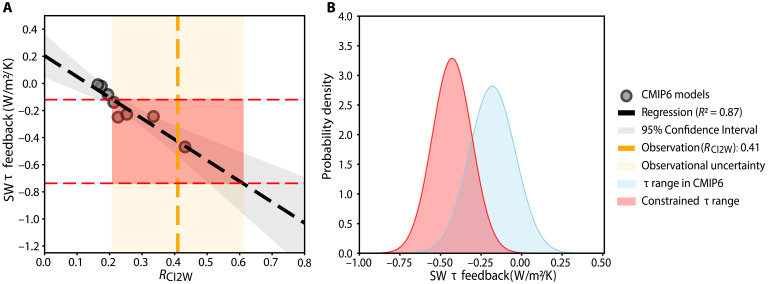
Emergent constraint on the SW cloud optical depth (τ) feedback. (**A**) Scatterplot showing the emergent relationship between *R*_CI2W_ (*x* axis) and the SW τ feedback (W m^−2^ K^−1^; *y* axis) across CMIP6 models (gray circles, *N* = 8). The black dashed line denotes the best-fit linear regression (*R*^2^ = 0.87, *P* < 0.001). Gray shading around the regression line indicates the standard prediction error (Eq. 5), representing uncertainty in the regression relationship. The observational reference from DARDAR-MODIS is shown by the vertical orange dashed line, with its SE indicated by orange shading. Horizontal dashed lines mark the resulting constrained estimate of the SW τ feedback. (**B**) Probability density functions (PDFs) of the SW τ feedback. The constrained posterior distribution (pink shading), calculated following Eq. 9, is shown alongside the unconstrained prior distribution from the CMIP6 model ensemble (blue shading).

Together, these results demonstrate both a robust physical linkage and a clear spatial correspondence between *R*_CI2W_ and the SW τ feedback in climate models. Across models, the correlation coefficient between *R*_CI2W_ and SW τ feedback reaches −0.93 (*P* < 0.001) (Fig. 4A). On the basis of this relationship and satellite-derived *R*_CI2W_ observations, we apply an emergent constraint framework to constrain the SW τ feedback in CMIP6 models (see Materials and Methods).

Because uncertainty in *R*_CI2W_ observations propagates into constraints on both the SW τ feedback and climate sensitivity, we first quantify the uncertainty in *R*_CI2W_ as 0.41 ± 0.20 using a Monte Carlo approach based on the observational uncertainties in ice and liquid water paths from DARDAR-MODIS. Accounting for this uncertainty, the SW τ feedback is revised from its original value of −0.18 ± 0.14 to −0.43 ± 0.12 W m^−2^ K^−1^ (Fig. 4B). This corrected estimate is comparable to, although slightly weaker than, constraints derived independently from MODIS (−0.58 ± 0.17 W m^−2^ K^−1^) and CloudSat (−0.46 ± 0.16 W m^−2^ K^−1^) (fig. S6).

**Fig. 5.**
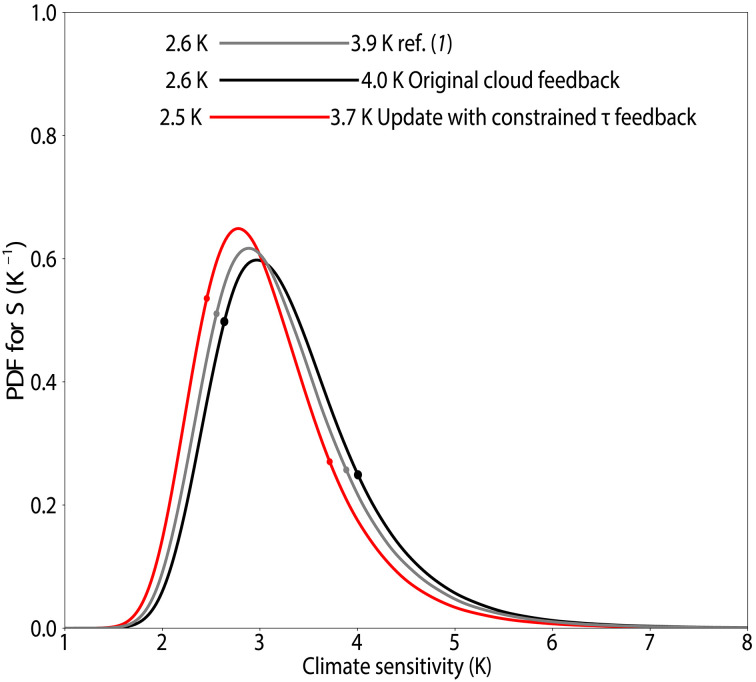
Updated climate sensitivity distributions after applying the observational constraint on the SW cloud optical depth (τ) feedback. The black curve shows the climate sensitivity distribution based on (*1*), while the gray curve represents the distribution derived from the original CMIP6 multimodel mean cloud feedback. The red curve shows the updated distribution after constraining the SW τ feedback using observed *R*_CI2W_. The likely (66%; 17th to 83rd percentile) range is indicated at the top, with circles marking the 17th and 83rd percentiles.

After correcting the τ feedback using DARDAR-MODIS observations, the total cloud feedback is reduced from 0.54 ± 0.29 to 0.30 ± 0.37 W m^−2^ K^−1^. We then incorporate the revised cloud feedback into a Bayesian framework to reestimate climate sensitivity, drawing on multiple lines of evidence (see Materials and Methods) (*1*). As shown in Fig. 5, the very likely (5 to 95%) range of climate sensitivity shifts from [2.3 to 4.7 K] to [2.2 to 4.5 K], while the likely (17 to 83%) range narrows from [2.6 to 4.0 K] to [2.5 to 3.7 K]. The median estimate decreases from 3.2 to 3.0 K, consistent with the CloudSat-based constraint and slightly higher than the MODIS-based estimate (2.9 K) (fig. S7). Together, these results suggest that biases in *R*_CI2W_ across the full cloud column lead to an underestimation of the SW τ feedback in current climate models, which in turn contributes to an overly positive total cloud feedback and an overestimation of climate sensitivity.

## DISCUSSION

Our analysis reveals that CMIP6 models generally underestimate *R*_CI2W_ throughout cloud layers, leading to a systematic underestimation of the SW cloud optical depth (τ) feedback. This ensemble-mean *R*_CI2W_ bias is primarily driven by insufficient simulated cloud ice, which outweighs the contribution from liquid water biases (figs. S8 and S9). Vertical profile analyses further confirm a pronounced ice deficit in the mid- to upper troposphere (fig. S10). This deficit is likely to influence τ feedback estimates through two mechanisms (fig. S11). First, across most of the troposphere, models simulate a decrease in ice and an increase in liquid water under warming; this ice-to-liquid phase transition enhances τ (*35*). Second, at upper levels, increases in ice concentration raise the IWP; once IWP exceeds a critical threshold [~200 g m^−2^ (*16*), corresponding to τ > 9.4 (*11*)], and the radiative effect of high-level ice clouds shifts from warming to cooling.

The historical “ice deficit” therefore limits both the amount of ice available for phase transitions and the likelihood that upper-level IWP reaches the threshold required for a cooling effect. As a result, the negative SW τ feedback is underestimated in current climate models. This underestimation contributes to an overly positive total cloud feedback and, consequently, an amplified warming response to CO_2_ doubling. Applying an observational constraint based on DARDAR-MODIS *R*_CI2W_ substantially strengthens the SW τ feedback and reduces the median climate sensitivity from 3.2 to 3.0 K. This constrained estimate suggests a more moderate climate sensitivity than implied by the unconstrained model ensemble. We emphasize that this result is based on an emergent constraint and is therefore subject to observational uncertainties and the representativeness of model physics, rather than constituting a direct measurement of the feedback.

To evaluate observational dependence, we repeated the analysis using MODIS and CloudSat observations. Both datasets independently confirm low *R*_CI2W_ values and weak SW τ feedback in the current models (figs. S3 and S10). The SW τ feedback is corrected to −0.58 ± 0.17 W m^−2^ K^−1^ using MODIS and −0.46 ± 0.16 W m^−2^ K^−1^ using CloudSat, both stronger than the active-passive combined estimate (−0.43 ± 0.12 W m^−2^ K^−1^). When equally weighting and averaging the constraints from all three datasets, the SW τ feedback is revised to −0.49 ± 0.26 W m^−2^ K^−1^. This indicates that observational uncertainties and interdataset differences broaden the confidence interval but do not substantially alter the constrained mean value. Overall, despite observational differences, all datasets consistently indicate that SW τ feedback is underestimated in current climate models.

Accounting for these corrections, the likely (17 to 83%) range of climate sensitivity shifts from the original [2.6 to 4.0] K to [2.4 to 3.6] K using MODIS and [2.4 to 3.7] K using CloudSat (fig. S11). Correspondingly, the median climate sensitivity decreases from 3.2 to 2.9 K (MODIS) and 3.0 K (CloudSat). These results demonstrate that, notwithstanding observational uncertainties, the central conclusion is robust: Current climate models tend to underestimate the strength of the SW τ feedback and overestimate climate sensitivity. The resulting more moderate sensitivity is consistent with conclusions drawn from other independent lines of evidence related to cloud feedbacks (*15*, *36*).

It should also be noted that the satellite retrievals used here—with IWP derived from active sensors and LWP from passive retrievals—are subject to systematic biases. Active sensors tend to underestimate IWP due to signal attenuation in precipitating conditions (*37*, *38*), whereas passive microwave algorithms typically overestimate LWP by misclassifying rainwater emission as cloud liquid water (*39*). Together, these limitations are expected to bias the retrievals toward higher LWP and lower IWP relative to their true values. Because CMIP6 models already underestimate *R*_CI2W_ relative to these observations, accounting for such observational biases would likely further widen the model-observation discrepancy, suggesting an even more pronounced underestimation of SW τ feedback in CMIP6 models due to their ice deficit.

This ice deficit persists regardless of which frozen hydrometeor categories are included in the evaluation. Even when snow and graupel are explicitly included, CMIP6 models still substantially underestimate total frozen condensate (*40*), demonstrating that the underestimated negative cloud feedback reflects a robust model bias rather than an artifact of hydrometeor categorization.

An additional source of uncertainty arises from the limited number of models included in the analysis, which could influence the linear relationship between *R*_CI2W_ and SW τ feedback used for the constraint. To assess the statistical robustness of this relationship, we applied leave-one-out cross-validation (LOOCV), which demonstrates strong out-of-sample predictive skill [*r* = 0.90, *P* = 2.0 × 10^−3^; root mean square error (RMSE) = 0.062 W m^−2^ K^−1^]. This result indicates that the inferred relationship is not driven by any single model and has substantial predictive capability. Nevertheless, expanding the model sample—particularly by including more models with satellite simulator outputs—would further strengthen future constraints.

We therefore recommend that future phases of CMIP encourage modeling groups to provide the variables required for such analyses, especially satellite simulator outputs from ISCCP and MODIS to better quantify SW τ feedback. Continued improvements in satellite retrievals of the ice-liquid water partitioning will also be critical for refining observational constraints and advancing understanding of cloud feedback processes in a warming climate.

## MATERIALS AND METHODS

### CMIP6 model outputs

We selected models from CMIP6 that provide outputs of cloud IWP (“clivi”) and total cloud water path (“clwvi”) to assess their performance in simulating the *R*_CI2W_. Model outputs from the “historical” experiment (1985–2014, ensemble member r1i1p1f1) were used in this study. Given that previous studies have verified the consistency of these models’ water paths with observations (*41*), *R*_CI2W_ serves as a metric for this analysis.

To evaluate different components of cloud feedback, we analyzed the “amip” and “amip-4K” experiments. The latter represents a uniform 4K surface warming scenario, while the former serves as the unperturbed control. The required variables include the following: cloud area fraction from the International Satellite Cloud Climatology Project (“clisccp”), surface downwelling clear-sky SW radiation (“rsdscs”), surface upwelling clear-sky SW radiation (“rsuscs”), and near-surface air temperature (“tas”). Eight CMIP6 models provided all the required variables, and their details are listed in table S2.

### Satellite observations of *R*_CI2W_

To validate CMIP6 simulations with accurate *R*_CI2W_ estimates, we exploited multisensor advantages to build a combined satellite dataset (hereafter DARDAR-MODIS). Specifically, we integrated MODIS passive sensor retrievals for LWP with DARDAR active sensor retrievals for IWP.

For the LWP, we used a merged global LWP dataset combining observations from multiple satellites. This dataset uses the MODIS level 3 monthly cloud products (MCD06COSP_M3_MODIS) as the global observational framework (*42*). However, MODIS systematically underestimates LWP over low-latitude ocean regions (*43*, *44*), particularly in precipitating and convective cloud systems (*45*–*48*). To address this bias, we applied corrections by replacing these underestimated values with the higher estimates derived from the Multi-sensor Advanced Climatology of Liquid Water Path (MAC-LWP) dataset (*49*). Since microwave sensors (used in MAC-LWP) can penetrate clouds and provide all-weather measurements, they are optimal for LWP retrieval (*50*). By integrating MODIS’s global coverage with MAC-LWP’s superior accuracy over oceans, we generated an improved global liquid water path dataset.

For the IWP, we used the DARDAR (radar/lidar) product. This product was developed to retrieve ice cloud properties globally from CloudSat and CALIPSO measurements using a specific universal parameterization of the particle size distribution (*51*) and the Varcloud optimal estimation algorithm (*31*, *32*). Therefore, DARDAR can detect thin cirrus clouds and the vertical structure of thick clouds and identify the ice in the whole air column (*52*–*54*), which has been widely evaluated with aircraft in situ measurements (*52*, *55*, *56*). Consequently, DARDAR is widely regarded as the best available IWP estimate (*57*, *58*). DARDAR data from 2007 to 2010 were used in this study. The retrieved variables have a horizontal resolution of 1.4 km and a vertical resolution of 60 m. To minimize contamination from precipitating clouds, we filtered the data using the DARMASK_Simplified_Categorization index provided in the DARDAR product. By selecting only pixels with a value of 1, we retained pure ice clouds and excluded those containing rain or supercooled liquid water (flags 5, 7, and 12 to 14). The effective radius of these ice particle ranges from 10 to 100 μm (*52*). In CMIP6 models, the definition of clivi either lacks an explicit size range or uses an autoconversion threshold of 100 μm for the clivi-to-snow transition (*40*, *59*). This size compatibility ensures that this observational dataset serves as an appropriate reference for evaluating modeled clivi.

For comparison purposes, we also used datasets based individually on MODIS and CloudSat observations. The MODIS dataset is derived from MODIS level 3 monthly cloud product (MCD06COSP_M3_MODIS), spanning from 2003 to 2014 to align with the final year of the CMIP6 historical simulations. The MCD06COSP product combines cloud property retrievals from both the Aqua and Terra instruments and is specifically designed to support climate studies that integrate modeling and observations (*42*). The CloudSat dataset is sourced from the CloudSat and CALIPSO Ice Cloud Property Product (2C-ICE) and the Radar-Visible Optical Depth Cloud Water Content product (2B-CWC-RVOD). Specifically, cloud IWP data are sourced from 2C-ICE (*60*, *61*), while liquid water path is obtained from 2B-CWC-RVOD, which offers a lower rate of missing data compared to the Radar-Only Cloud Water Content product (2B-CWC-RO) (*62*–*64*). Because of a battery anomaly in April 2011, CloudSat data are only available for the period 2007–2010.

### Calculation of SW τ feedback

We quantified the cloud amount feedback, cloud altitude feedback, and cloud τ feedback using cloud radiative kernels (*11*–*13*) and outputs from the ISCCP simulator (*65*, *66*). To calculate the τ feedback, we first derived the anomaly in the distribution of cloud fractions caused by a unit change in temperature ($\Delta C_{p\tau }$, unit: %/K) (*67*–*69*)

$$\Delta C_{p\tau }=\left(\frac{C_{\mathrm{p\tau}}}{C_{\text{tot}}}\right)\Delta C_{\text{tot}}+\Delta {C^{*}}_{p\tau }$$ (1)

where $C_{p\tau }$ refers to the cloud fraction in a bin (*p*, τ) defined by cloud top pressure (CTP) and τ, and $C_{\text{tot}}$ is the total cloud cover, with $\Delta C_{\text{tot}}$ representing the change in total cloud fraction. The first term on the right-hand side represents the contribution to $\Delta C_{p\tau }$ from a hypothetical change in total cloud cover distributed across CTP-τ categories while keeping the original normalized distribution unchanged. The second term on the right-hand side represents the change in the cloud distribution in CTP and τ while keeping the total cloud fraction unchanged.

Next, we calculate the component of the cloud radiative kernel deviation ($K_{p\tau }^{\prime }$) that independently contributes to the radiative effect due to τ ($K_{\tau }^{\prime }$, unit: W m^−2^ %^−1^)

$$K_{\tau }^{\prime }=\sum_{p=1}^{P}\left(K_{p\tau }^{\prime }\sum_{\tau =1}^{T}\frac{C_{p\tau }}{C_{\text{tot}}}\right)$$ (2)

Here, $K_{p\tau }^{\prime }$ represents the deviation of the cloud radiative kernel for each bin from its weighted average value. By weighting $K_{p\tau }^{\prime }$ with cloud fraction distributions to nullify CTP effects, we obtain $K_{\tau }^{\prime }$, which purely captures the radiative sensitivity to τ variations.

Last, we obtain the τ-induced radiation anomalies (denoted $f_{\tau }$, unit: W m^−2^ K^−1^) by multiplying this cloud radiative kernel ($K_{\tau }^{\prime }$) by the change in cloud fraction due to only τ

$$f_{\tau }=\sum_{\tau =1}^{T}\left(K_{\tau }^{\prime }\sum_{p=1}^{P}\Delta {C^{*}}_{p\tau }\right)$$ (3)

### Quantifying the error in *R*_CI2W_ observations

We used a Monte Carlo approach to quantifying the observational uncertainty in *R*_CI2W_. This method is widely recognized as a robust and reliable technique for uncertainty propagation in complex or nonlinear functions and has been successfully applied to quantify uncertainty in climate data records from Earth observation (*70*, *71*). Specifically, for each climatological grid point, we performed 2000 sampling iterations wherein the multiyear mean IWP and LWP were independently perturbed according to their observational uncertainties. *R*_CI2W_ was recalculated for each perturbed sample, yielding a distribution of climatological *R*_CI2W_ values. The SD of this distribution represents the propagated observational uncertainty of the climatological *R*_CI2W_.

### Emergent constraint on SW τ feedback

The emergent constraint method exploits an empirical near-linear relationship between a historical observable (here, x,*R*_CI2W_) and a future climate response (here, $\hat{f_{\tau }}$, SW τ feedback) across model ensembles (*72*–*74*). By projecting the observed *R*_CI2W_ with its uncertainty (represented by one SD) onto the $\hat{f_{\tau }}$ axis through the empirical linear relationship, we obtain a constrained estimate of future SW τ feedback with reduced uncertainty. We establish the emergent constraint relationship using least-squares linear regression (Eq. 4) (*75*), with prediction error [$\sigma_{\hat{f_{\tau }}\left(x\right)}$] calculated by Eq. 5

$${\hat{f_{\tau }}}_{i}=a\;x_{i}+b$$ (4)

where ${\hat{f_{\tau }}}_{i}$ is the value corresponding to $x_{i}$; and a and b are the slope and intercept, respectively

$$\sigma_{\hat{f_{\tau }}\left(x\right)}=s\sqrt{1+\frac{1}{N}+\frac{{\left(x-\bar{x}\right)}^{2}}{N\sigma_{x}^{2}}}$$ (5)

Here, s represents the least-squares error that is to be minimized, which is calculated via Eq. 6. *N* denotes the total number of models within the ensemble. Furthermore, σ_*x*_ is the variance of the variable x (determined by Eq. 7), and $\bar{x}$ is its corresponding mean value

$$s^{2}=\frac{1}{N-2}\sum_{n=1}^{N}{\left(\hat{f_{\tau }}-{\hat{f_{\tau }}}_{i}\right)}^{2}$$ (6)

$$\sigma_{x}=\sqrt{\frac{\sum_{n=1}^{N}{\left(x_{i}-\bar{x}\right)}^{2}}{N}}$$ (7)

### Calculation of probability density function

We used Eq. 8 to estimate the probability density function (PDF) of the original $\hat{f_{\tau }}$ before applying the emergent constraint (*75*–*77*)

$$\text{PDF}\left(\hat{f_{\tau }}/x\right)=\frac{1}{\sqrt{2\pi \cdot \sigma_{\hat{f_{\tau }}}^{2}}}\text{exp}\left\{-\frac{{\left[\hat{f_{\tau }}-f\left(x\right)\right]}^{2}}{2\sigma_{\hat{f_{\tau }}}^{2}}\right\}$$ (8)

where $\text{PDF}\left(\hat{f_{\tau }}/x\right)$ is the PDF around the best-fit linear regression, representing the PDF of $\hat{f_{\tau }}$ given *x*. After the constraint is applied, the PDF for the constrained projected variable $\text{PDF}\left(\hat{f_{\tau }}/\text{ob}\right)$ is calculated by numerically integrating $\text{PDF}\left(\hat{f_{\tau }}/\text{ob}\right)$ and PDF(ob) (Eq. 9), where $\text{PDF}\left(\hat{f_{\tau }}/\text{ob}\right)$ is the probability density for the $f_{\tau }$ given the observable *R*_CI2W_, and PDF(ob) is the observation-based PDF for the historical observable *R*_CI2W_

$$\text{PDF}\left(\hat{f_{\tau }}\right)=\int_{-\infty }^{+\infty }\text{PDF}\left(\hat{f_{\tau }}/\text{ob}\right)\cdot \text{PDF}\left(\text{ob}\right)\cdot d\;\text{ob}$$ (9)

### Cross-validation of the emergent constraint

To assess the robustness of the emergent constraint, we applied LOOCV (*72*, *78*, *79*). In this approach, we iteratively excluded one model from the ensemble, rederived the regression relationship using the remaining models, and used it to predict the excluded model’s feedback. This procedure was repeated for all models to evaluate out-of-sample predictive skill. The correlation between LOOCV predictions and actual model values, along with RMSE, quantifies the constraint’s robustness.

### Reestimation of climate sensitivity

We reestimate climate sensitivity using a Bayesian approach, which allows us to generate a PDF for climate sensitivity by integrating multiple lines of evidence—namely, feedback process understanding, the historical climate record, and the paleoclimate record [as referenced in (*1*)]. The calculation follows Bayes’ theorem

$$p\left(S|E\right)=\frac{p\left(E|S\right)p\left(S\right)}{p\left(E\right)}$$ (10)

Here, S represents climate sensitivity and *E* denotes the evidence. The term p(S∣E) is the posterior probability density of *S* conditional on the evidence. On the right-hand side, p(E∣S) is the likelihood, which quantifies how probable the evidence *E* is for any given value of S. p(S) is the prior distribution of S, reflecting our knowledge before considering the new evidence. p(E) serves as a normalization constant to ensure the total probability equals one.

In this study, the updated SW cloud optical depth feedback ($f_{\tau }$) is treated as new evidence to update the posterior distribution of 𝑆. The likelihood p(E∣S) reflects how consistent various values of S are with the observed constraint on $f_{\tau }$. All other feedback parameters and terms derived from historical and paleoclimate records remain unchanged in this estimation.

## Supplementary Materials

### This PDF file includes:

Figs. S1 to S11 Tables S1 to S3 References
