# Supplementary material for: Observational constraints from global ice-phase fraction indicate moderate climate sensitivity
Source: Sci Adv. 2026 Jun 5;12(23):eaea0731. doi: 10.1126/sciadv.aea0731 (PMC13240212; doi:10.1126/sciadv.aea0731)
Supplement: Supplementary file 1 — Figs. S1 to S11 Tables S1 to S3 References [file sciadv.aea0731_sm.pdf]

Science Advances
AAAS

Supplementary Materials for

Observational constraints from global ice-phase fraction indicate moderate climate sensitivity

Rui Zhou *et al.*

Corresponding author: Tingfeng Dou, doutf@ucas.ac.cn; Ivy Tan, ivy.tan@colorado.edu; Cunde Xiao, cdxiao@bnu.edu.cn

Sci. Adv. **12**, eaea0731 (2026)
DOI: 10.1126/sciadv.aea0731

This PDF file includes:

Figs. S1 to S11
Tables S1 to S3
References

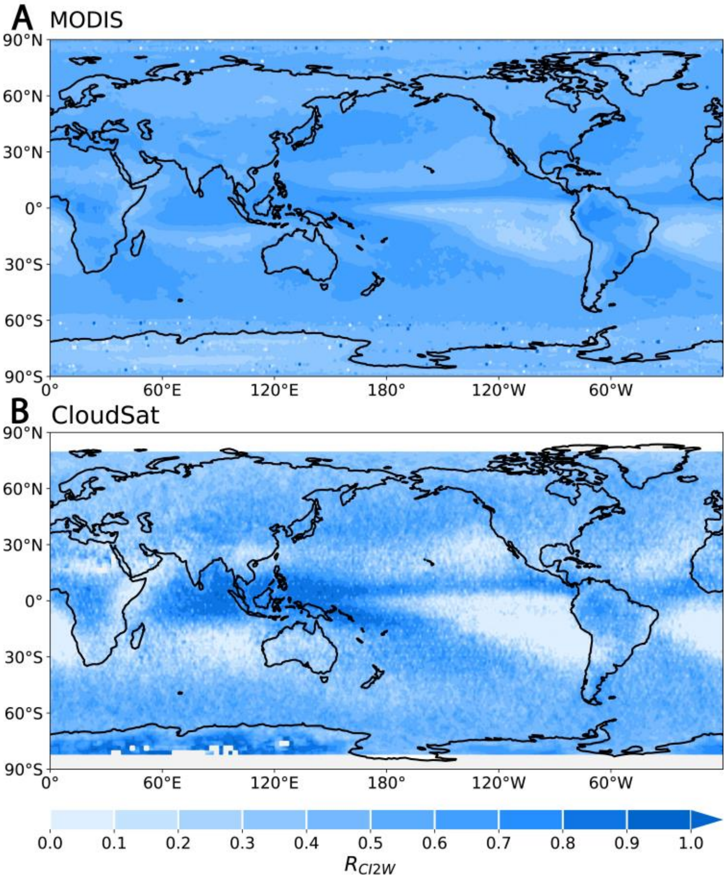

Fig. S1. Observed spatial distribution of the Cloud Ice-to-Water Ratio (R_{CI2W}) from two independent satellite products. (A) Passive-sensor-based observations from MODIS. (B) Active-sensor-based observations from CloudSat.

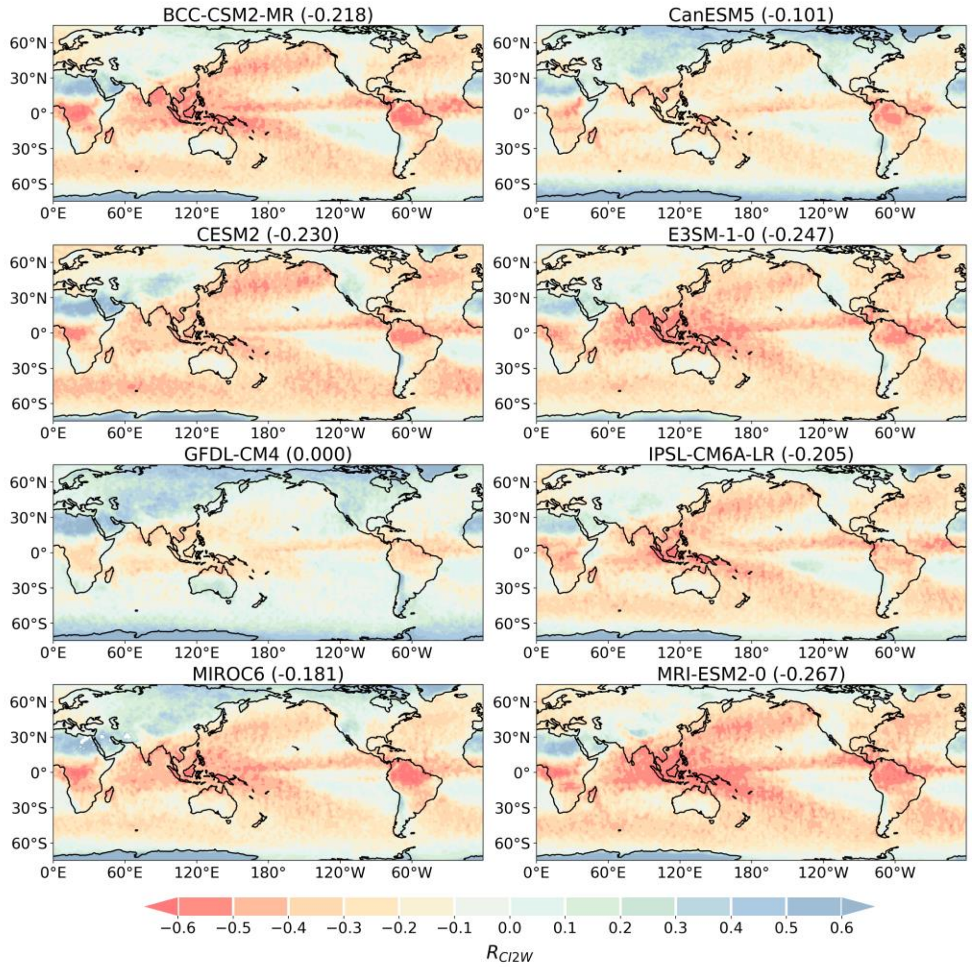

Fig. S2. Absolute simulation bias in the Cloud Ice-to-Water Ratio (R_{CI2W}) relative to DARDAR–MODIS observations across CMIP6 models. The values in parentheses indicate the global mean bias.

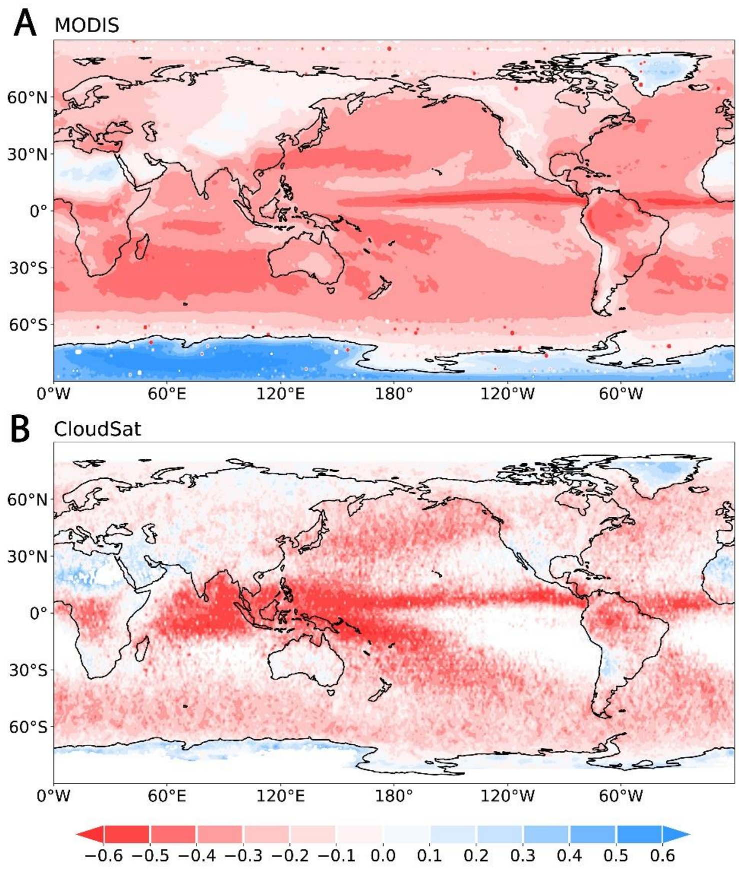

Fig. S3. Simulation bias of the CMIP6 multi-model mean Cloud Ice-to-Water Ratio (R_{CI2W}) relative to satellite observations. (A) Bias relative to MODIS observations. (B) Bias relative to CloudSat observations. Negative values indicate regions where R_{CI2W} is underestimated by the models.

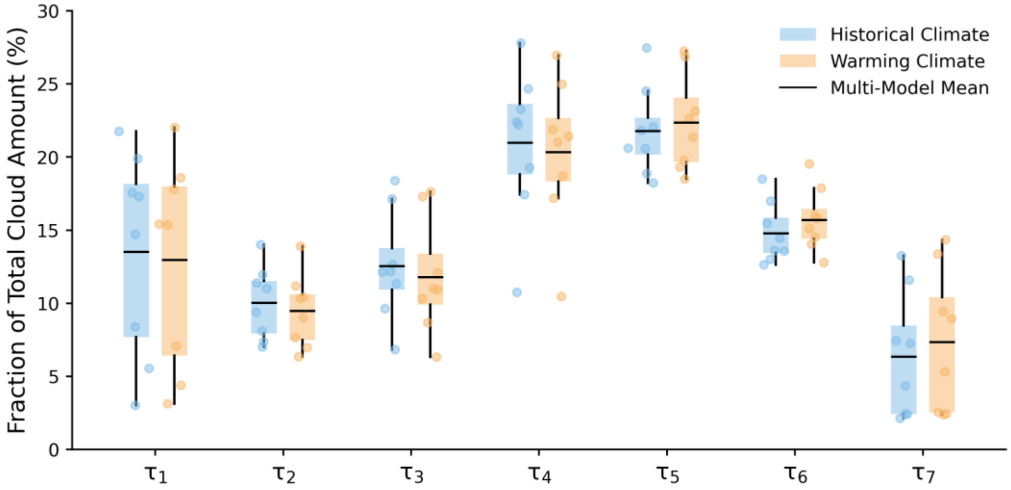

Fig. S4. Comparison of cloud fraction across cloud optical depth (τ) bins under historical and 4 K warming climates. Box-and-whisker plots show cloud fraction distributions under historical (blue) and warming (orange) climates across seven τ bins (τ_1–τ_7), defined as: τ_1 = 0–0.3, τ_2 = 0.3–1.3, τ_3 = 1.3–3.6, τ_4 = 3.6–9.4, τ_5 = 9.4–23, τ_6 = 23–60, and τ_7 = 60–380. Each box plot represents the multi-model ensemble distribution, with the central black line indicating the multi-model mean and overlaid dots showing individual model results. Box boundaries indicate the 25th and 75th percentiles, whiskers extend to 1.5 times the interquartile range.

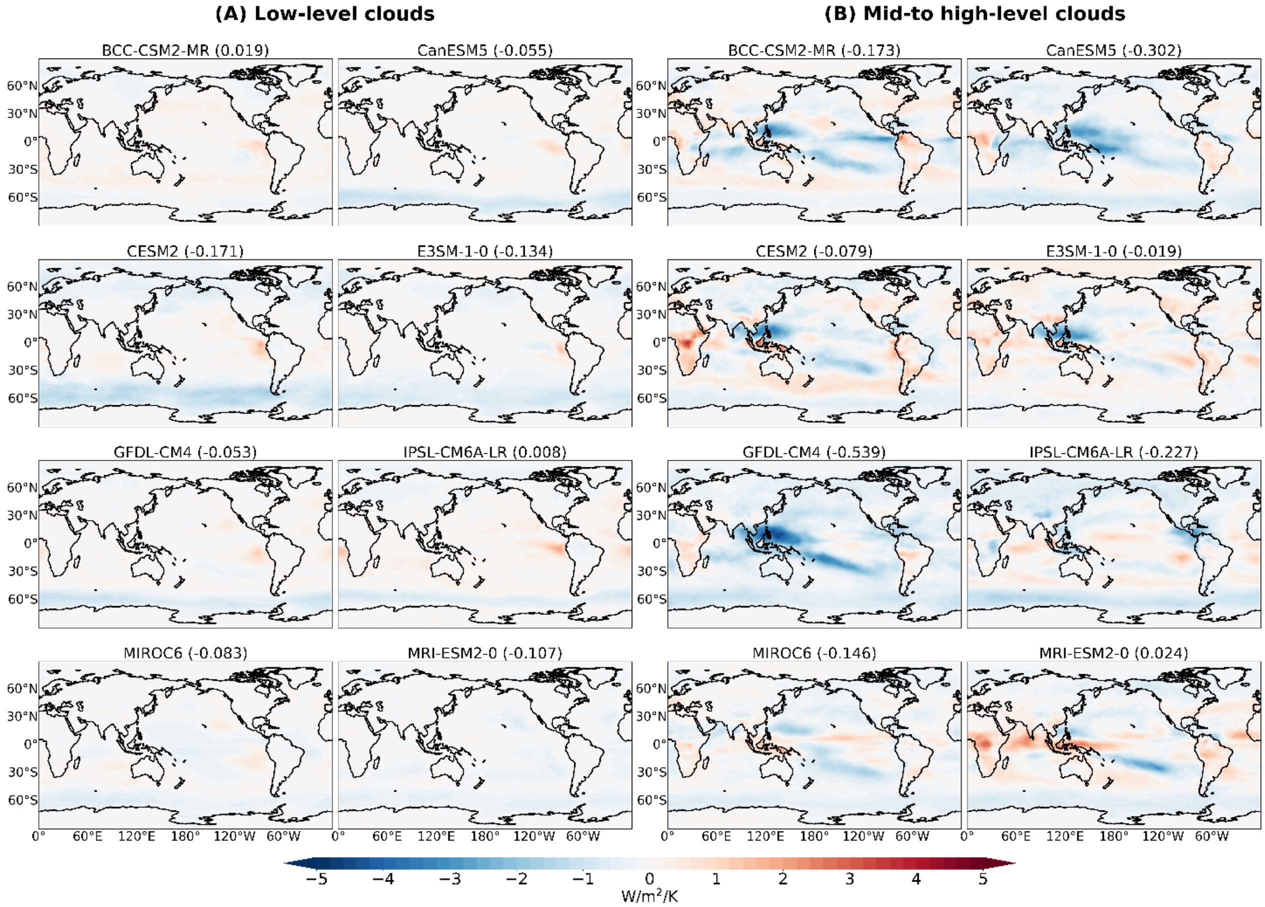

Fig. S5. Global spatial distribution of the shortwave cloud optical depth (τ) feedback by cloud vertical regime. Maps show the SW τ feedback for (A) low-level clouds (cloud-top pressure > 680 hPa) and (B) mid- to high-level clouds (cloud-top pressure ≤ 680 hPa) across the CMIP6 model ensemble. The values in parentheses indicate the global area-weighted mean feedback.

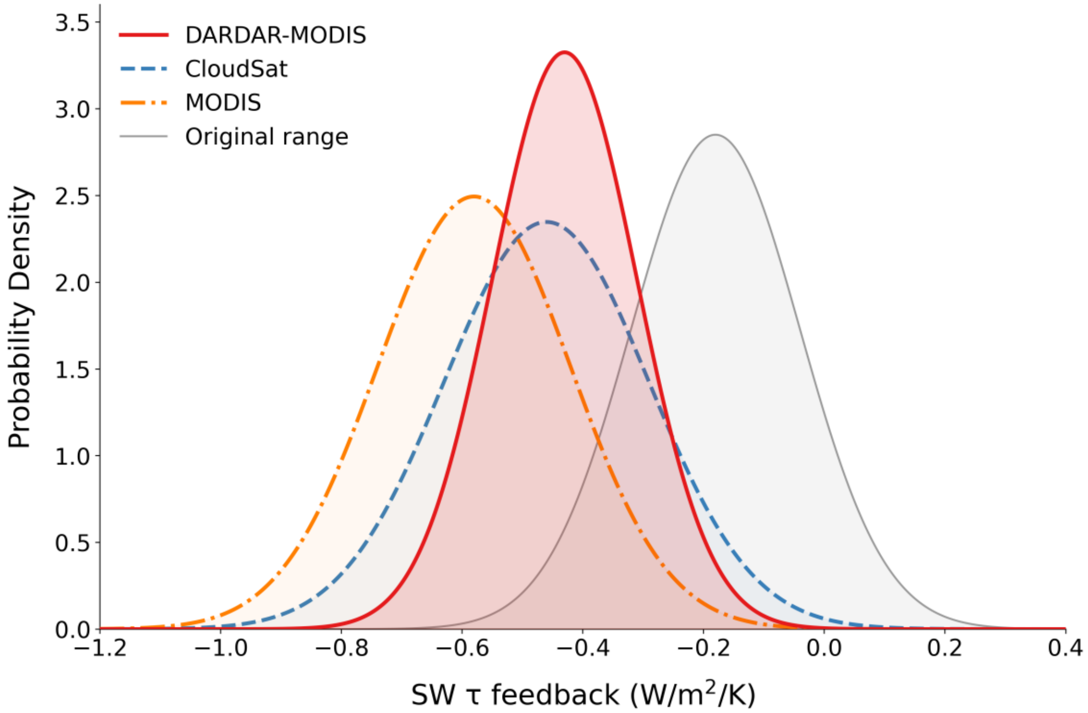

Fig. S6. Comparison of observational constraints on the shortwave cloud optical depth (SW τ) feedback derived from different satellite datasets. Probability density functions (PDFs) show constraints based on multi-sensor fusion (DARDAR–MODIS; red solid line with shading), active observations (CloudSat; blue dashed line), and passive observations (MODIS; orange dash–dot line). The grey solid line represents the PDF of the original unconstrained CMIP6 model ensemble. All three observational constraints consistently indicate a stronger negative SW τ feedback than the original model range.

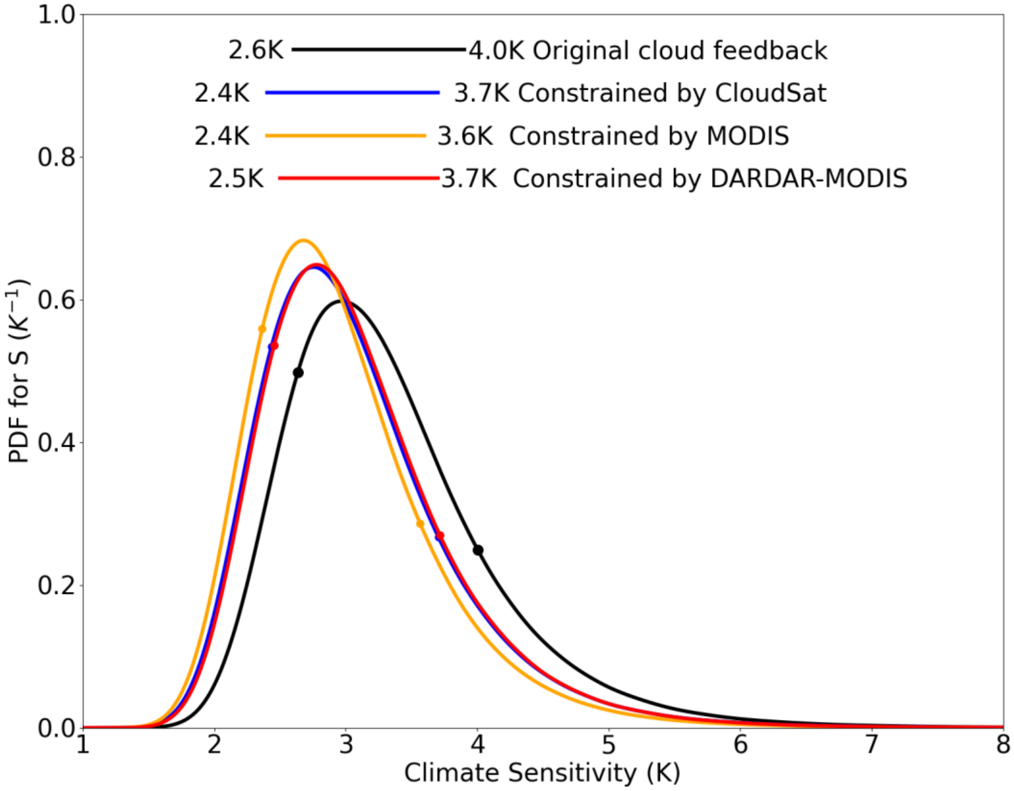

Fig. S7. Updated climate sensitivity distributions after applying different observational constraints on the shortwave cloud optical depth (SW τ) feedback. The black curve shows the distribution derived from the original CMIP6 multi-model mean cloud feedback. Colored curves show the updated distributions after constraining the SW τ feedback using Cloud Ice-to-Water Ratio estimates from different observational datasets: CloudSat (blue), MODIS (orange), and DARDAR–MODIS (red). The likely (66%; 17th–83rd percentile) range for each distribution is indicated at the top left, with circles on the curves marking the corresponding 17th and 83rd percentiles.

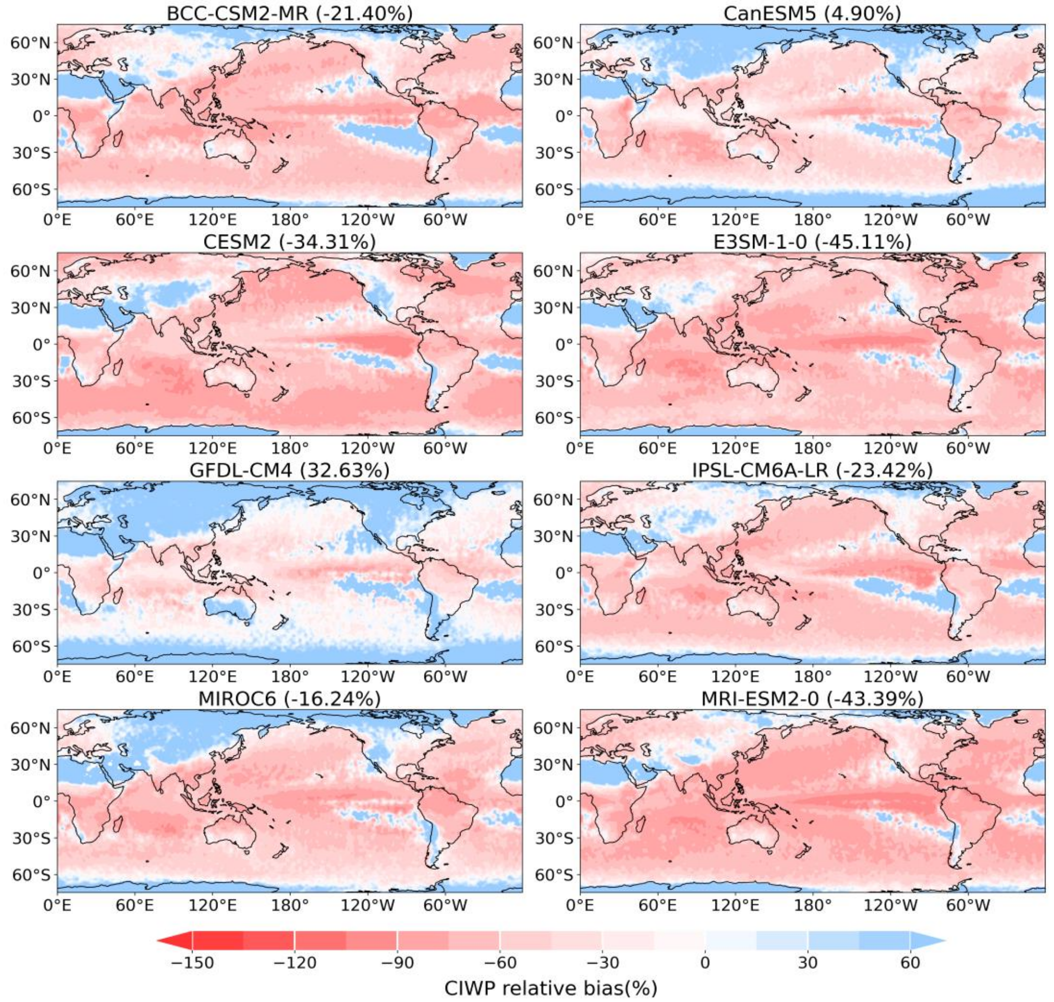

Fig. S8. Relative bias in cloud ice water path across CMIP6 models relative to DARDAR–MODIS observations. The values in parentheses indicate the global mean bias.

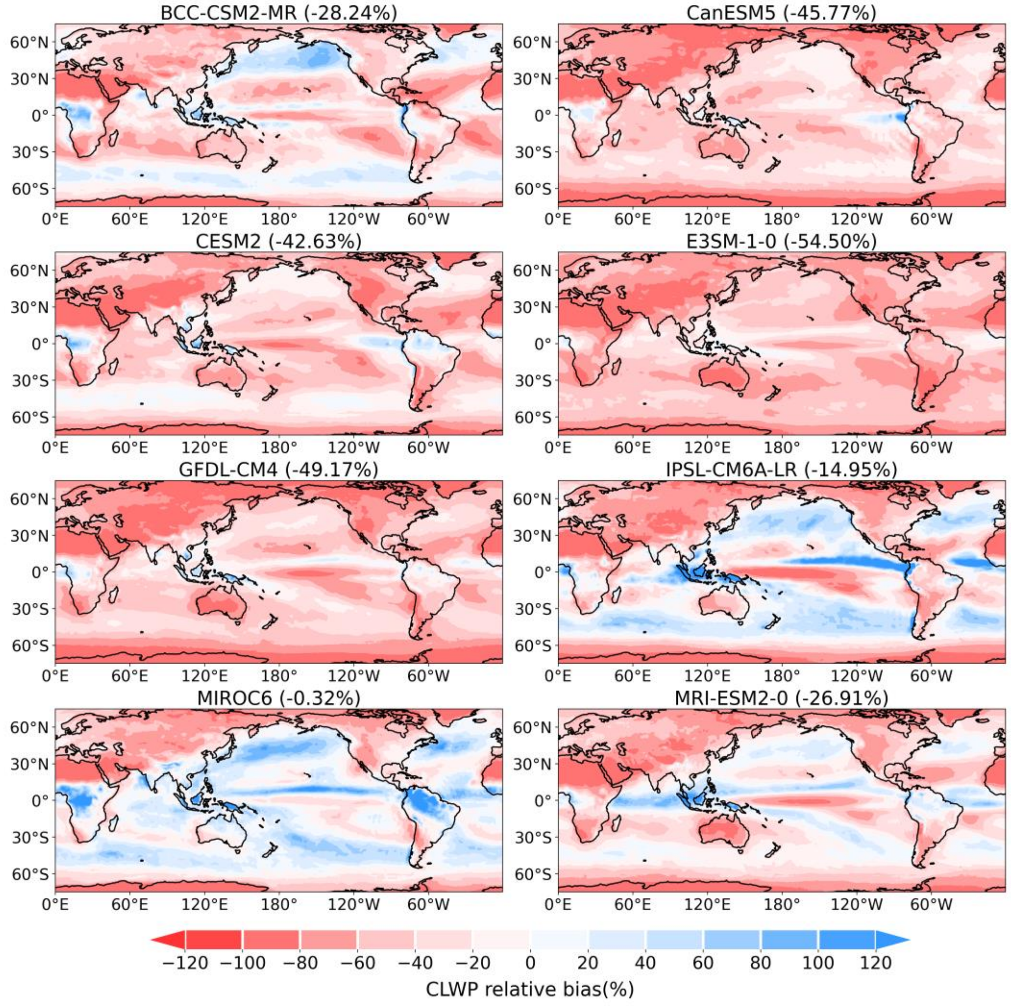

Fig. S9. Relative bias in cloud liquid water path across CMIP6 models relative to DARDAR–MODIS observations. The values in parentheses indicate the global mean bias.

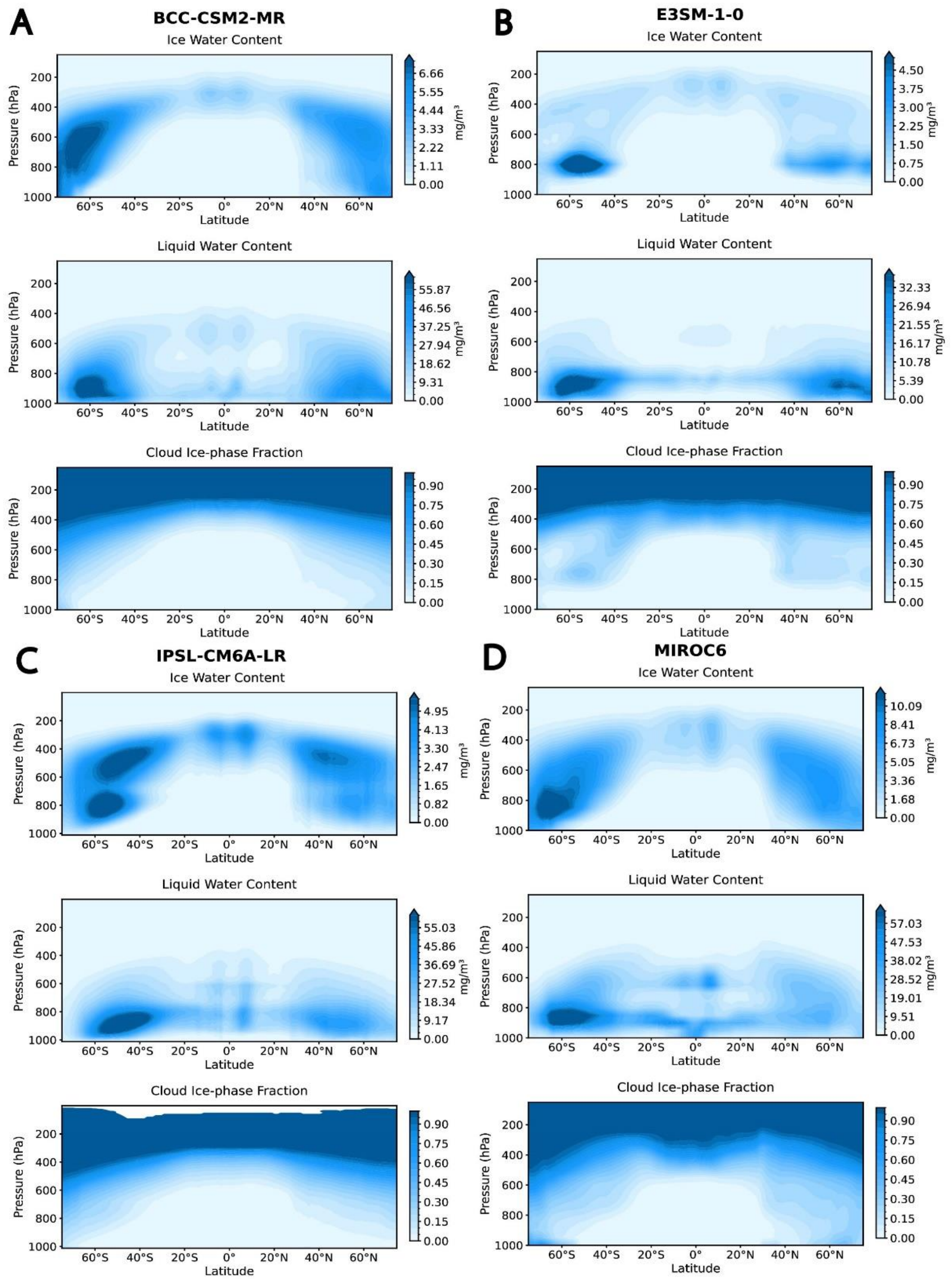

Fig. S10. Zonal-mean vertical distributions of cloud properties under the historical scenario for four CMIP6 models. Panels show results for (A) BCC-CSM2-MR, (B) E3SM-1-0, (C) IPSL-CM6A-LR, and (D) MIROC6. For each model, the sub-panels (from top to bottom) display cloud ice content, cloud liquid water content, and cloud ice-phase fraction. Corresponding observational references are shown in Fig. 2 of ref. (*80*).

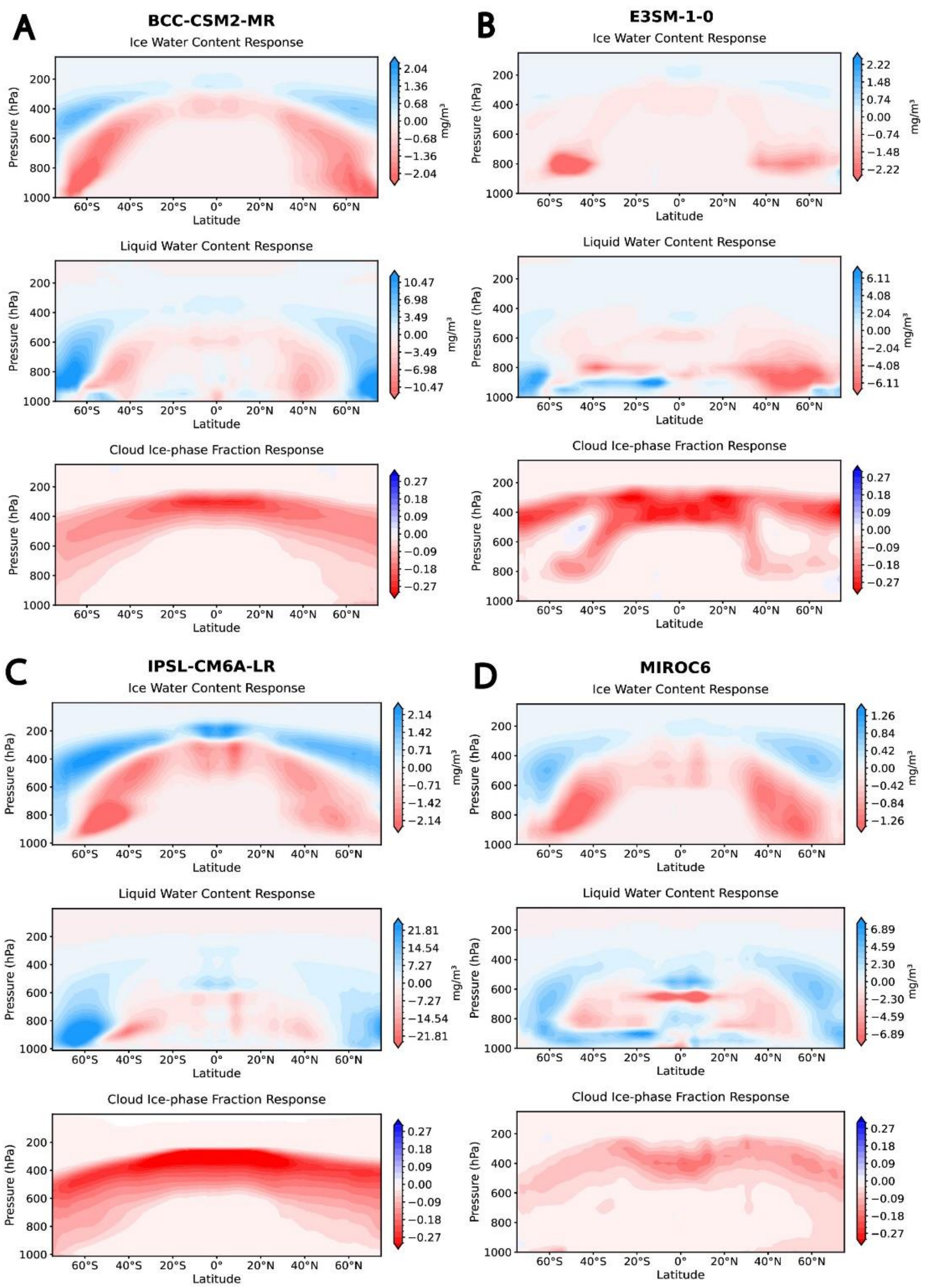

Fig. S11. Zonal-mean responses of cloud properties to warming (abrupt-4×CO_2 minus historical) for four CMIP6 models. Panels show results for (A) BCC-CSM2-MR, (B) E3SM-1-0, (C) IPSL-CM6A-LR, and (D) MIROC6. For each model, the sub-panels (from top to bottom) display the responses of cloud ice content, cloud liquid water content, and cloud ice-phase fraction.

Table S1. Overview of observational constraints on different components of cloud feedbacks.

Cloud feedback type	Region	Observation	Original cloud feedback ($W m^{-2} K^{-1}$)	Constrained Cloud Feedback ($W m^{-2} K^{-1}$)	Likely climate sensitivity range (Median)	Reference
High-cloud altitude feedback	Global	CALIPSO	/	0.20 ± 0.21	/	(*18*)
Marine low-level cloud feedback	Tropical	CloudSat-CALIPSO	/	0.56 ± 0.15	3.1–3.8 K (3.5 K)	(*14*)
	Mid-low latitude	MODIS	0.37 ± 0.37	0.19 ± 0.12	2.4–3.6 K (3.0 K)	(*15*)
Anvil cloud area feedback	Tropical	DARDAR-Cloud and 2C-ICE	0.20 ± 0.21	0.03 ± 0.06	2.8–4.2 K (3.4 K)	(*16*)
	Tropical	CloudSat-CALIPSO and CERES		0.02 ± 0.07	/	(*17*)
Cloud optical depth feedback	Global	MODIS	–0.18 ± 0.14	–0.58 ± 0.17	2.4–3.6 K (2.9 K)	This study
		CloudSat		–0.46 ± 0.16	2.4–3.7 K (3.0 K)	
		DARDAR–MODIS		–0.43 ± 0.12	2.5–3.7 K (3.0 K)	

Table S2. Information on the climate models used to calculate the shortwave cloud optical depth (SW τ) feedback.

	Model name	Resolution (lat×lon)	Institution
1	BCC-CSM2-MR	160×320	Beijing Climate Center, China
2	CanESM5	64× 128	Canadian Centre for Climate Modelling and Analysis, Canada
3	CESM2	192 × 288	National Center for Atmospheric Research, Climate and Global Dynamics Laboratory, USA
4	E3SM-1-0	90 x 90	The United States Department of Energy (DOE), USA
5	GFDL-CM4	180× 288	National Oceanic and Atmospheric Administration, Geophysical Fluid Dynamics Laboratory, USA
6	IPSL-CM6A-LR	143 × 144	Institute Pierre Simon Laplace, France
7	MIROC6	128 × 256	Atmosphere and Ocean Research Institute, The University of Tokyo, Japan
8	MRI-ESM2-0	160×320	Meteorological Research Institute, Japan

Table S3. Values of cloud ice-to-water ratio (R_{CI2W}) and shortwave cloud optical depth feedback (SW τ feedback) for the CMIP6 models shown in Figure 4.

	Model name	R_{CI2W}	SW τ feedback ($W\ m^{-2}\ K^{-1}$)
1	BCC-CSM2-MR	0.213	−0.14
2	CanESM5	0.335	−0.244
3	CESM2	0.195	−0.082
4	E3SM-1-0	0.175	−0.021
5	GFDL-CM4	0.432	−0.469
6	IPSL-CM6A-LR	0.226	−0.249
7	MIROC6	0.253	−0.228
8	MRI-ESM2-0	0.164	−0.009

REFERENCES

1. S. C. Sherwood, M. J. Webb, J. D. Annan, K. C. Armour, P. M. Forster, J. C. Hargreaves, G. Hegerl, S. A. Klein, K. D. Marvel, E. J. Rohling, M. Watanabe, T. Andrews, P. Braconnot, C. S. Bretherton, G. L. Foster, Z. Hausfather, A. S. Von Der Heydt, R. Knutti, T. Mauritsen, J. R. Norris, C. Proistosescu, M. Rugenstein, G. A. Schmidt, K. B. Tokarska, M. D. Zelinka, An assessment of Earth's climate sensitivity using multiple lines of evidence. *Rev. Geophys.* **58**, e2019RG000678 (2020).

2. G. A. Meehl, C. A. Senior, V. Eyring, G. Flato, J.-F. Lamarque, R. J. Stouffer, K. E. Taylor, M. Schlund, Context for interpreting equilibrium climate sensitivity and transient climate response from the CMIP6 Earth system models. *Sci. Adv.* **6**, eaba1981 (2020).

3. R. Knutti, G. C. Hegerl, The equilibrium sensitivity of the Earth's temperature to radiation changes. *Nat. Geosci.* **1**, 735–743 (2008).

4. G. L. Stephens, Cloud feedbacks in the climate system: A critical review. *J. Climate* **18**, 237–273 (2005).

5. T. Zhou, X. Chen, M. Zuo, J. Jiang, Earth's climate sensitivity: Methods, climate feedback processes, progresses and prospects. *Quat. Sci.* **43**, 604–624 (2023).

6. N. G. Loeb, D. R. Doelling, H. Wang, W. Su, C. Nguyen, J. G. Corbett, L. Liang, C. Mitrescu, F. G. Rose, S. Kato, Clouds and the Earth's Radiant Energy System (CERES) Energy Balanced and Filled (EBAF) Top-of-Atmosphere (TOA) edition-4.0 data product. *J. Clim.* **31**, 895–918 (2018).

7. A. I. Flores-Anderson, J. Cardille, K. Azad, E. Cherrington, Y. Zhang, S. Wilson, Spatial and temporal availability of cloud-free optical observations in the tropics to monitor deforestation. *Sci. Data* **10**, 550 (2023).

8. M. D. Shupe, J. M. Intrieri, Cloud radiative forcing of the arctic surface: The influence of cloud properties, surface albedo, and solar zenith angle. *J. Climate* **17**, 616–628 (2004).

9. A. Gettelman, S. C. Sherwood, Processes responsible for cloud feedback. *Curr. Clim. Change Rep.* **2**, 179–189 (2016).

10. G. Cesana, T. Storelvmo, Improving climate projections by understanding how cloud phase affects radiation. *J. Geophys. Res. Atmos.* **122**, 4594–4599 (2017).

11. M. D. Zelinka, S. A. Klein, D. L. Hartmann, Computing and partitioning cloud feedbacks using cloud property histograms. Part I: Cloud radiative kernels. *J. Climate* **25**, 3715–3735 (2012).

12. M. D. Zelinka, S. A. Klein, D. L. Hartmann, Computing and partitioning cloud feedbacks using cloud property histograms. Part II: Attribution to changes in cloud amount, altitude, and optical depth. *J. Climate* **25**, 3736–3754 (2012).

13. C. Zhou, M. D. Zelinka, A. E. Dessler, P. Yang, An analysis of the short-term cloud feedback using MODIS data. *J. Climate* **26**, 4803–4815 (2013).

14. G. V. Cesana, A. D. Del Genio, Observational constraint on cloud feedbacks suggests moderate climate sensitivity. *Nat. Clim. Chang.* **11**, 213–218 (2021).

15. T. A. Myers, R. C. Scott, M. D. Zelinka, S. A. Klein, J. R. Norris, P. M. Caldwell, Observational constraints on low cloud feedback reduce uncertainty of climate sensitivity. *Nat. Clim. Chang.* **11**, 501–507 (2021).

16. A. B. Sokol, C. J. Wall, D. L. Hartmann, Greater climate sensitivity implied by anvil cloud thinning. *Nat. Geosci.* **17**, 398–403 (2024).

17. B. McKim, S. Bony, J.-L. Dufresne, Weak anvil cloud area feedback suggested by physical and observational constraints. *Nat. Geosci.* **17**, 392–397 (2024).

18. C. Zhou, A. E. Dessler, M. D. Zelinka, P. Yang, T. Wang, Cirrus feedback on interannual climate fluctuations. *Geophys. Res. Lett.* **41**, 9166–9173 (2014).

19. M. D. Zelinka, T. A. Myers, D. T. McCoy, S. Po-Chedley, P. M. Caldwell, P. Ceppi, S. A. Klein, K. E. Taylor, Causes of higher climate sensitivity in CMIP6 models. *Geophys. Res. Lett.* **47**, e2019GL085782 (2020).

20. N. D. Gordon, S. A. Klein, Low-cloud optical depth feedback in climate models. *J. Geophys. Res. Atmos.* **119**, 6052–6065 (2014).

21. C. R. Terai, S. A. Klein, M. D. Zelinka, Constraining the low-cloud optical depth feedback at middle and high latitudes using satellite observations. *J. Geophys. Res. Atmos.* **121**, 9696–9716 (2016).

22. I. Tan, T. Storelvmo, M. D. Zelinka, Observational constraints on mixed-phase clouds imply higher climate sensitivity. *Science* **352**, 224–227 (2016).

23. I. Tan, L. Oreopoulos, N. Cho, The role of thermodynamic phase shifts in cloud optical depth variations with temperature. *Geophys. Res. Lett.* **46**, 4502–4511 (2019).

24. C. J. Wall, T. Storelvmo, J. R. Norris, I. Tan, Observational constraints on Southern Ocean cloud-phase feedback. *J. Climate* **35**, 5087–5102 (2022).

25. S. Hofer, L. C. Hahn, J. K. Shaw, Z. S. McGraw, O. Bruno, F. Hellmuth, M. Pietschnig, I. A. Mostue, R. O. David, T. Carlsen, T. Storelvmo, Realistic representation of mixed-phase clouds increases projected climate warming. *Commun. Earth Environ.* **5**, 390 (2024).

26. I. Tan, T. Storelvmo, Sensitivity study on the influence of cloud microphysical parameters on mixed-phase cloud thermodynamic phase partitioning in CAM5. *J. Atmos. Sci.* **73**, 709–728 (2016).

27. H. Zhang, M. Zhao, Q. Chen, Q. Wang, S. Zhao, X. Zhou, J. Peng, Water and ice cloud optical thickness changes and radiative effects in East Asia. *J. Quant. Spectrosc. Radiat. Transf.* **254**, 107213 (2020).

28. Y. Huang, X. Dong, J. E. Kay, B. Xi, E. A. McIlhattan, The climate response to increased cloud liquid water over the Arctic in CESM1: A sensitivity study of Wegener–Bergeron–Findeisen process. *Climate Dynam.* **56**, 3373–3394 (2021).

29. J. S. Daniel, S. Solomon, R. W. Portmann, A. O. Langford, C. S. Eubank, E. G. Dutton, W. Madsen, Cloud liquid water and ice measurements from spectrally resolved near-infrared observations: A new technique. *J. Geophys. Res. Atmos.* **107**, 4599 (2002).

30. J. Huang, P. Minnis, B. Lin, Y. Yi, T.-F. Fan, S. Sun-Mack, J. K. Ayers, Determination of ice water path in ice-over-water cloud systems using combined MODIS and AMSR-E measurements. *Geophys. Res. Lett.* **33**, L21801 (2006).

31. J. Delanoë, R. J. Hogan, A variational scheme for retrieving ice cloud properties from combined radar, lidar, and infrared radiometer. *J. Geophys. Res. Atmos.* **113**, D07204 (2008).

32. J. Delanoë, R. J. Hogan, Combined CloudSat-CALIPSO-MODIS retrievals of the properties of ice clouds. *J. Geophys. Res. Atmos.* **115**, D00H29 (2010).

33. G. L. Stephens, M. A. Smalley, M. D. Lebsock, The cloudy nature of tropical rains. *J. Geophys. Res. Atmos.* **124**, 171–188 (2019).

34. R. M. Schulte, M. D. Lebsock, J. M. Haynes, What CloudSat cannot see: Liquid water content profiles inferred from MODIS and CALIOP observations. *Atmos. Meas. Tech.* **16**, 3531–3546 (2023).

35. C. J. Wall, D. Paynter, Y. Qin, M. Debolskiy, M. L. Duffy, T. Michibata, B. M. Duran, N. J. Lutsko, P.-L. Ma, B. Medeiros, T. Storelvmo, M. Zhao, Decomposing cloud radiative feedbacks by cloud-top phase. *J. Climate* **38**, 4023–4043 (2025).

36. I. Tan, C. Zhou, A. Lamy, C. L. Stauffer, Moderate climate sensitivity due to opposing mixed-phase cloud feedbacks. *NPJ Clim. Atmos. Sci.* **8**, 86 (2025).

37. D. E. Waliser, J.-L. F. Li, C. P. Woods, R. T. Austin, J. Bacmeister, J. Chern, A. Del Genio, J. H. Jiang, Z. Kuang, H. Meng, P. Minnis, S. Platnick, W. B. Rossow, G. L. Stephens, S. Sun-

Mack, W.-K. Tao, A. M. Tompkins, D. G. Vane, C. Walker, D. Wu, Cloud ice: A climate model challenge with signs and expectations of progress. *J. Geophys. Res. Atmos.* **114**, D00A21 (2009).

38. D. I. Duncan, P. Eriksson, An update on global atmospheric ice estimates from satellite observations and reanalyses. *Atmos. Chem. Phys.* **18**, 11205–11219 (2018).

39. C. W. O'Dell, F. J. Wentz, and R. Bennartz, Cloud liquid water path from satellite-based passive microwave observations: A new climatology over the global oceans. *J. Climate* **21**, 1721–1739 (2008).

40. P. Eriksson, A. Baró Pérez, N. Müller, H. Hallborn, E. May, M. Brath, S. A. Buehler, L. Ickes, Advancements and continued challenges in observations and global modelling of atmospheric ice mass. *Atmos. Chem. Phys.* **26**, 2741–2768 (2026).

41. A. Lauer, L. Bock, B. Hassler, M. Schröder, M. Stengel, Cloud climatologies from global climate models—A comparison of CMIP5 and CMIP6 models with satellite data. *J. Climate* **36**, 281–311 (2023).

42. R. Pincus, P. A. Hubanks, S. Platnick, K. Meyer, R. E. Holz, D. Botambekov, C. J. Wall, Updated observations of clouds by MODIS for global model assessment. *Earth Syst. Sci. Data* **15**, 2483–2497 (2023).

43. D. Painemal, P. Zuidema, Assessment of MODIS cloud effective radius and optical thickness retrievals over the Southeast Pacific with VOCALS-REx in situ measurements. *J. Geophys. Res. Atmos.* **116**, D24206 (2011).

44. H. Cho, Z. Zhang, K. Meyer, M. Lebsock, S. Platnick, A. S. Ackerman, L. Di Girolamo, L. C.-Labonnote, C. Cornet, J. Riedi, R. E. Holz, Frequency and causes of failed MODIS cloud property retrievals for liquid phase clouds over global oceans. *J. Geophys. Res. Atmos.* **120**, 4132–4154 (2015).

45. C. Seethala, Á. Horváth, Global assessment of AMSR-E and MODIS cloud liquid water path retrievals in warm oceanic clouds. *J. Geophys. Res. Atmos.* **115**, D13202 (2010).

46. M. Lebsock, H. Su, Application of active spaceborne remote sensing for understanding biases between passive cloud water path retrievals. *J. Geophys. Res. Atmos.* **119**, 8962–8979 (2014).

47. R. E. Holz, S. Platnick, K. Meyer, M. Vaughan, A. Heidinger, P. Yang, G. Wind, S. Dutcher, S. Ackerman, N. Amarasinghe, F. Nagle, C. Wang, Resolving ice cloud optical thickness biases between CALIOP and MODIS using infrared retrievals. *Atmos. Chem. Phys.* **16**, 5075–5090 (2016).

48. S. R. Noble, J. G. Hudson, MODIS comparisons with northeastern Pacific in situ stratocumulus microphysics. *J. Geophys. Res. Atmos.* **120**, 8332–8344 (2015).

49. G. S. Elsaesser, C. W. O'Dell, M. D. Lebsock, R. Bennartz, T. J. Greenwald, F. J. Wentz, The Multi-Sensor Advanced Climatology of Liquid Water Path (MAC-LWP). *J. Climate* **30**, 10193–10210 (2017).

50. G. L. Stephens, C. D. Kummerow, The remote sensing of clouds and precipitation from space: A review. *J. Atmos. Sci.* **64**, 3742–3765 (2007).

51. J. M. E. Delanoë, A. J. Heymsfield, A. Protat, A. Bansemer, R. J. Hogan, Normalized particle size distribution for remote sensing application. *J. Geophys. Res. Atmos.* **119**, 4204–4227 (2014).

52. Y. Hong, G. Liu, The characteristics of ice cloud properties derived from CloudSat and CALIPSO measurements. *J. Climate* **28**, 3880–3901 (2015).

53. K. Sassen, Z. Wang, D. Liu, Global distribution of cirrus clouds from CloudSat/Cloud-Aerosol Lidar and Infrared Pathfinder Satellite Observations (CALIPSO) measurements. *J. Geophys. Res. Atmos.* **113**, D00A12 (2008).

54. M. S. Kulie, L. Milani, N. B. Wood, S. A. Tushaus, R. Bennartz, T. S. L'Ecuyer, A shallow cumuliform snowfall census using spaceborne radar. *J. Hydrometeorol.* **17**, 1261–1279 (2016).

55. M. Deng, G. G. Mace, Z. Wang, R. P. Lawson, Evaluation of several A-train ice cloud retrieval products with in situ measurements collected during the SPARTICUS campaign. *J. Appl. Meteorol. Climatol.* **52**, 1014–1030 (2013).

56. M. Saito, H. Iwabuchi, P. Yang, G. Tang, M. D. King, M. Sekiguchi, Ice particle morphology and microphysical properties of cirrus clouds inferred from combined CALIOP-IIR measurements. *J. Geophys. Res. Atmos.* **122**, 4440–4462 (2017).

57. T. H. M. Stein, J. Delanoë, R. J. Hogan, A comparison among four different retrieval methods for ice-cloud properties using data from CloudSat, CALIPSO, and MODIS. *J. Appl. Meteorol. Climatol.* **50**, 1952–1969 (2011).

58. S. Eliasson, S. A. Buehler, M. Milz, P. Eriksson, V. O. John, Assessing observed and modelled spatial distributions of ice water path using satellite data. *Atmos. Chem. Phys.* **11**, 375–391 (2011).

59. H. Morrison, A. Gettelman, A new two-moment bulk stratiform cloud microphysics scheme in the Community Atmosphere Model, version 3 (CAM3). Part I: Description and numerical tests. *J. Climate* **21**, 3624–3649 (2008).

60. M. Deng, G. G. Mace, Z. Wang, E. Berry, CloudSat 2C-ICE product update with a new Z_e parameterization in lidar-only region. *J. Geophys. Res. Atmos.* **120**, 12198–12208 (2015).

61. J.-L. F. Li, K.-M. Xu, J. H. Jiang, W.-L. Lee, L.-C. Wang, J.-Y. Yu, G. Stephens, E. Fetzer, Y.-H. Wang, An overview of CMIP5 and CMIP6 simulated cloud ice, radiation fields, surface wind stress, sea surface temperatures, and precipitation over tropical and subtropical oceans. *J. Geophys. Res. Atmos.* **125**, e2020JD032848 (2020).

62. L. Yu, Y. Fu, Y. Yang, X. Pan, R. Tan, Trumpet-shaped topography modulation of the frequency, vertical structures, and water path of cloud systems in the summertime over the southeastern Tibetan Plateau: A perspective of daytime–nighttime differences. *J. Geophys. Res. Atmos.* **125**, e2019JD031803 (2020).

63. Level 2B Radar-Visible Optical Depth Cloud Water Content (2B-CWC-RVOD) Process Description Document (ResearchGate, 2008); https://researchgate.net/publication/255657913_Level_2B_Radar-

Visible_Optical_Depth_Cloud_Water_Content_2B-CWC-RVOD_Process_Description_Document.

64. R. Seto, T. Koike, S. Kanae, Representing cloud water content of extensive cloud systems over land using satellite-based passive microwave observations with a coupled land and atmosphere assimilation method. *J. Geophys. Res. Atmos.* **123**, 12829–12856 (2018).

65. S. A. Klein, C. Jakob, Validation and sensitivities of frontal clouds simulated by the ECMWF model. *Mon. Weather Rev.* **127**, 2514–2531 (1999).

66. M. Webb, C. Senior, S. Bony, J.-J. Morcrette, Combining ERBE and ISCCP data to assess clouds in the Hadley Centre, ECMWF and LMD atmospheric climate models. *Climate Dynam.* **17**, 905–922 (2001).

67. M. D. Zelinka, S. A. Klein, K. E. Taylor, T. Andrews, M. J. Webb, J. M. Gregory, P. M. Forster, Contributions of different cloud types to feedbacks and rapid adjustments in CMIP5. *J. Climate* **26**, 5007–5027 (2013).

68. M. D. Zelinka, S. A. Klein, Y. Qin, T. A. Myers, Evaluating climate models' cloud feedbacks against expert judgment. *J. Geophys. Res. Atmos.* **127**, e2021JD035198 (2022).

69. M. Zelinka, mzelinka/assessed-cloud-fbks: 28 September 2023 Release, Zenodo (2023); https://zenodo.org/records/8387963.

70. Evaluation of measurement data—Supplement 1 to the "Guide to the expression of uncertainty in measurement"—(2008); https://doi.org/10.59161/JCGM101-2008.

71. C. J. Merchant, F. Paul, T. Popp, M. Ablain, S. Bontemps, P. Defourny, R. Hollmann, T. Lavergne, A. Laeng, G. de Leeuw, J. Mittaz, C. Poulsen, A. C. Povey, M. Reuter, S. Sathyendranath, S. Sandven, V. F. Sofieva, W. Wagner, Uncertainty information in climate data records from Earth observation. *Earth Syst. Sci. Data* **9**, 511–527 (2017).

72. A. Hall, P. Cox, C. Huntingford, S. Klein, Progressing emergent constraints on future climate change. *Nat. Clim. Chang.* **9**, 269–278 (2019).

73. V. Eyring, P. M. Cox, G. M. Flato, P. J. Gleckler, G. Abramowitz, P. Caldwell, W. D. Collins, B. K. Gier, A. D. Hall, F. M. Hoffman, G. C. Hurtt, A. Jahn, C. D. Jones, S. A. Klein, J. P. Krasting, L. Kwiatkowski, R. Lorenz, E. Maloney, G. A. Meehl, A. G. Pendergrass, R. Pincus, A. C. Ruane, J. L. Russell, B. M. Sanderson, B. D. Santer, S. C. Sherwood, I. R. Simpson, R. J. Stouffer, M. S. Williamson, Taking climate model evaluation to the next level. *Nat. Clim. Chang.* **9**, 102–110 (2019).

74. C. W. Thackeray, A. Hall, An emergent constraint on future Arctic sea-ice albedo feedback. *Nat. Clim. Chang.* **9**, 972–978 (2019).

75. P. M. Cox, C. Huntingford, M. S. Williamson, Emergent constraint on equilibrium climate sensitivity from global temperature variability. *Nature* **553**, 319–322 (2018).

76. M. Schlund, A. Lauer, P. Gentine, S. C. Sherwood, V. Eyring, Emergent constraints on equilibrium climate sensitivity in CMIP5: Do they hold for CMIP6? *Earth Syst. Dynam.* **11**, 1233–1258 (2020).

77. Y. Chai, G. Martins, C. Nobre, C. von Randow, T. Chen, H. Dolman, Constraining Amazonian land surface temperature sensitivity to precipitation and the probability of forest dieback. *NPJ Clim. Atmos. Sci.* **4**, 6 (2021).

78. A. Vehtari, A. Gelman, J. Gabry, Practical Bayesian model evaluation using leave-one-out cross-validation and WAIC. *Stat Comput* **27**, 1413–1432 (2017).

79. A. Ribes, S. Qasmi, N. P. Gillett, Making climate projections conditional on historical observations. *Sci. Adv.* **7**, eabc0671 (2021).

80. L. Huang, J. H. Jiang, Z. Wang, H. Su, M. Deng, S. Massie, Climatology of cloud water content associated with different cloud types observed by A-Train satellites. *J. Geophys. Res. Atmos.* **120**, 4196–4212 (2015).
